# Influence of Equilibration Time and Bull-Specific Extender for Cryopreservation on Semen Quality and Fertility in German Holstein Friesian Bulls: A Controlled Field Trial

**DOI:** 10.3390/ani13142285

**Published:** 2023-07-12

**Authors:** Laura Pieper, Tristan Meschede, Markus Jung, Ulrich Janowitz, Martin Schulze

**Affiliations:** 1 Institute for Reproduction of Farm Animals Schönow, Bernauer Allee 10, D-16321 Bernau, Germany; m.jung@ifn-schoenow.de; 2 Rinder-Union West eG, Schifffahrter Damm 235a, D-48147 Muenster, Germany; tmeschede@ruweg.de (T.M.); ujanowitz@ruweg.de (U.J.)

**Keywords:** bovine, equilibration time, cryopreservation, extender, fertility, sperm quality

## Abstract

**Simple Summary:**

Cattle sperm needs to be equilibrated to reduce damage during freezing. Equilibration is the time that the sperm spends in a freezing solution, the extender. Commonly, bull studs require 4 h equilibration time. However, this creates a high workload on the two to three days when bull sperm is collected during a week. We aimed to find the best equilibration time and extender, and to observe the effects on fertility in the field. We found that increasing the equilibration time from 4 h to 24 h improved sperm quality when using the extender Triladyl. The extenders Triladyl and OptiXcell yielded the best laboratory results at 24 h equilibration compared to the extender BioXcell. Field fertility did not differ among the three combinations of extender and equilibration time that we tested. Therefore, it is possible to change from 4 h to 24 h equilibration time while potentially improving laboratory sperm quality and without negative effects on fertility.

**Abstract:**

Equilibration with an extender is necessary to allow cryopreservation of bovine sperm. The aim of trial 1 was to assess the effect of 24 h versus 4 h equilibration time with three different extenders on sperm quality and to select the preferred extender for each bull. The aim of trial 2 was to investigate the effect of using a 24 h equilibration time with a bull-specific extender on field fertility. For trial 1, three ejaculates each from eight Holstein Friesian breeding bulls were used as the split-sample, including two equilibration times (4 h and 24 h) and three extenders (BioXcell, Triladyl, and OptiXcell). For trial 2, from 5 to 10 ejaculates from the same bulls were collected and treated (split-sample) as BioXcell with 4 h equilibration and either Triladyl or OptiXcell, both with 24 h equilibration. A total of 11,059 straws were used for insemination of cows and heifers. For Triladyl, progressive sperm motility, acrosome defects, and plasma membrane and acrosome integrity improved with a 24 h compared to a 4 h equilibration time. Four bulls each were used with Triladyl and OptiXcell for trial 2. In trial 2, non-return rates did not differ among groups. Therefore, using a 24 h equilibration time might improve in vitro sperm parameters, depending on the extender used. Moreover, it would be possible to change from 4 h to 24 h equilibration time without impairing field fertility.

## 1. Introduction

In order to distribute and store the genetic material of valuable individuals, it is necessary to freeze the semen of domestic and wild animal species. The main challenge herein is to avoid cell damage due to freezing and thawing. Extenders are commonly used to dilute semen to the desired concentration and add, for example, cryoprotectants, nutrients and antibiotics to nourish and preserve the sperm [1]. The time of contact between cryoprotectant and semen prior to freezing is called the equilibration time.

Some studies found very short equilibration times (<4 h) to be negatively associated with sperm motility and plasma membrane integrity after thawing [2,3,4]. On the other hand, further increasing the equilibration time was not or positively associated with the aforementioned laboratory parameters [3,5,6,7]. However, researchers were unable to show the influences of increasing equilibration times on fertility in the field [5,6,7]. A common practice in German commercial breeding bull centers is an equilibration time of 4 h prior to freezing. However, this requires semen to be frozen on the same day of semen collection, which might negatively affect the working schedule of laboratory personnel [3]. Therefore, some artificial insemination (AI) centers have changed from a 4 h to a 24 h equilibration time in order to freeze semen on the following day of semen collection and distribute the workload. The effects of changing equilibration time from 4 h to 24 h on laboratory parameters and field fertility have not been scientifically assessed in German Holstein Friesian bulls.

Different extenders with or without proteins of animal origin (e.g., egg yolk) have been studied previously using different equilibration times in bovine and other related species [3,4,7]. Triladyl is a commonly used egg yolk-based extender, whereas BioXcell (based on soybean lecithin) and OptiXcell (based on synthetic liposomes) do not use animal proteins. There is a trend against the use of extenders that utilize animal products due to concerns regarding disease transmission, hygiene and natural variation [1]. In a study by Bousseau et al. [8], relevant amounts of bacterial contamination were found in egg yolk (from whole eggs, liquid or powder) and prepared extender containing egg yolk, whereas a soybean–glycerol-based extender was sterile. Recent studies showed similar or superior laboratory results after thawing (e.g., sperm motility, plasma membrane and acrosome integrity, DNA fragmentation index or high mitochondrial membrane potential) when using OptiXcell compared to Triladyl [7,9].

Leaders of German breeding companies reported anecdotally that some bulls showed improved laboratory results with certain extenders, whereas others worsened their performance. Therefore, the lack of differences when changing extenders and equilibration times in terms of fertility in some previous studies might be due to these individual differences in bulls. Conversely, selecting a bull-specific extender and increasing the equilibration time to 24 h might improve field fertility.

The aim of trial 1 was to compare laboratory parameters when using 4 h or 24 h equilibration time with different extenders and to find the best semen extender for the 24 h equilibration time for each bull based on an extended spectrum of spermatological methods after thawing. The hypothesis was that laboratory parameters for each bull would differ based on the extenders used and that we could select a preferred extender for each bull.

The aim of trial 2 was to evaluate laboratory parameters and field fertility when using the selected bull-specific extender and a prolonged equilibration time of 24 h compared to 4 h. We hypothesized that bull-specific extenders with a prolonged equilibration time would result in higher field fertility than a 4 h equilibration time with a non-bull-specific extender.

## 2. Materials and Methods

### 2.1. Study Design

#### 2.1.1. Trial 1

Trial 1 was designed as a 2 × 3 factorial design using two equilibration times (4 h or 24 h) and three different extenders (BioXcell [IMV, L’Aigle, France], Triladyl [Minitube, Tiefenbach, Germany] or OptiXcell [IMV, L’Aigle, France]) in a split-sample procedure. The resulting treatments for 4 h and 24 h equilibration times were for BioXcell BIO4 and BIO24, respectively, for Triladyl TRI4 and TRI24, respectively, and for OptiXcell OPTX4 and OPTX24, respectively. Ejaculates from 8 Holstein Friesian bulls were collected three consecutive times in August 2020. After initial laboratory screening (see below), each ejaculate was divided into six portions which were used to produce at least 20 straws (0.23 mL each) for each of the treatment groups. Batches were frozen for at least 24 h in liquid nitrogen. After thawing, straws were subjected to laboratory assessment (see below). Based on these results, the best extender for each bull for the 24 h equilibration time was selected.

#### 2.1.2. Trial 2

Trial 2 was designed as a blinded controlled field trial, comparing BIO4 and either TRI24 or OPTX24, which corresponded to treatments in trial 1. Between November 2020 and July 2021, the aforementioned bulls were collected between 5 and 10 times and ejaculates were prepared using the split-sample procedure. All samples were divided into two equal portions and prepared as BIO4 and either as TRI24 or OPTX24 (depending on the bull). Samples were frozen and, after 24 h, three straws were thawed from each batch for laboratory assessment (see below). Laboratory technicians were not blinded to the treatment group. After a quarantine of 30 days, straws were distributed to 54 professional artificial insemination technicians who conducted inseminations in the regions North Rhine–Westphalia and Rhineland Palatinate in Germany on heifers and cows from commercial dairy farms using the study straws between December 2020 and April 2022. AI technicians and farmers were blinded to the treatment groups of the straws.

### 2.2. Sample Size Calculation

For trial 2, a sample size for repeated measures analysis and between factor comparison was calculated using G*Power (Version 3.1.9.6, Franz Faul, University of Kiel, Kiel, Germany). The expected means for non-return rates 60 (NRR60) from first inseminations for the three groups were 65% (BIO4), 72% (TRI24) and 75% (OPTX24) with an equal standard deviation of 5, but with twice as many animals in BIO4 than in TRI24 or OPTX24, resulting in an effect size of 0.589. The alpha level was set at 0.05 and power at 0.8. We expected an average of eight repetitions (ejaculates) within bulls and a correlation of 0.3 among ejaculates. Based on these assumptions, we calculated a minimum sample size of 15. This resulted in all eight bulls in BIO4 and four in TRI24 or OPTX24 each (total sample size of *n* = 16). With each bull providing approximately eight ejaculates, we expected to have 128 batches (ejaculate with the corresponding treatment). For each batch, we were aiming for at least 50 inseminations in order to avoid biased NRR60 due to low sample size. Therefore, we were aiming for at least 6400 inseminations.

### 2.3. Bull Center, Animals and Semen Collection

The participating AI center (Rinder-Union West, AI Station Borken) was located in the western part of Germany, kept on average 130 bulls and produced approximately 1.3 to 1.6 million straws per year. Eight Holstein Friesian breeding bulls (six black and white Holstein, two red Holstein) with a high expected usage for the following years were selected for the main trial. Age at enrollment for trial 1 ranged from 1.7 to 3.8 years and overall breeding values were superior to the average population (Table 1).

Ejaculates from bulls were collected twice weekly into sterile polypropylene vials (Model: Falcon, VWR International GmbH, Darmstadt, Germany) using a sterilized, pre-warmed artificial vagina (Danish Model, Minitube, Tiefenbach, Germany). Ejaculates were screened for volume, sperm concentration, sperm motility and morphology (see below).

### 2.4. Laboratory Evaluations Prior to Freezing

Volume of ejaculates was determined by weight using an electronic precision scale (Modell PCB 2000-1, Kern, Balingen, Germany). Ejaculates with a volume <2 mL were discarded. Native sperm concentration was measured photometrically (Photometer SDM 5, Minitube, Tiefenbach, Germany) by mixing 3.5 mL physiological saline solution with 70 µL of the ejaculate in a 4 mL measuring cuvette and measuring extinction at 546 nm wavelength. Ejaculates with a concentration <600,000 spermatozoa/mL were discarded.

Sperm motility was evaluated after dilution using Computer Assisted Sperm Analysis System (CASA, AndroVision, Minitube, Tiefenbach, Germany). Next, 20 µL native sperm and 20 µL physiological saline were mixed on a pre-warmed slide (38 °C) and covered with a cover slip. Progressive motility was assessed by the program in five fields of view. Ejaculates with progressive motility <70% were discarded.

For morphological assessment, 50 µL of the ejaculate was mixed with 1% phosphate-buffered formol solution to achieve 50 × 10^6^ to 100 × 10^6^ spermatozoa/mL. Samples were sent (at room temperature) to the Institute for Reproduction of Farm Animals Schönow (IFN, Bernau, Germany) for morphological assessment within 48 h of sample preparation. Samples were assessed using a phase contrast microscope and 800× magnification [10]. A total of 4 µL of the sample was pipetted onto a slide, then covered with cover slip, and at least 200 spermatozoa were assessed as either morphologically intact or as having morphological or acrosome alterations. Samples with >20% morphological or >10% acrosome alterations were discarded.

### 2.5. Preparation of Extenders and Equilibration

Native ejaculates were diluted in a sterile wide neck bottle (Model: Duran, Carl Roth, Karsruhe, Germany) using the extender according to the manufacturers’ instructions to achieve the desired concentration of 87 × 10^6^ motile spermatozoa/mL. The amount of extender needed was calculated using the computer program AndroVision (Minitube, Tiefenbach, Germany). For preparation of BioXcell, 250 mL of extender concentrate was diluted with 1000 mL bi-distilled, sterile water. For preparation of OptiXcell, 250 mL of extender concentrate was diluted with 500 mL of bi-distilled, sterile water. For the preparation of Triladyl, 250 mL extender concentrate was mixed with 750 mL of bi-distilled, sterile water. Then, fresh chicken eggs from agricultural trade (fit for human consumption) were acquired. Eggshells were flamed for disinfection, and yolk and egg white were separated manually. Yolks (250 mL) were filtered and mixed with the extender solution (yolk content = 20% in the final solution). For BioXcell and OptiXcell, dilution was performed in one single step, whereas for Triladyl, dilution was performed in two steps, 10 min apart from each other.

Batches BIO4, TRI4 and OPTX4 were filled into straws and, thereafter, equilibrated horizontally for 4 h at 4 °C. Batches BIO24, TRI24 and OPTX24 were equilibrated in a wide-neck bottle at 4 °C and, thereafter, filled into straws at the same temperature.

### 2.6. Packaging and Freezing

Straws (0.23 mL, Mini-Pailletten, Minitube, Tiefenbach, Germany) were filled with approximately 20 million motile spermatozoa to aim for at least 10 million motile sperm after thawing. Filling and printing of legally required information onto straws was performed using an automatic filling and printing machine (Modell MPP Quattro, Minitube, Tiefenbach, Germany). Identification of the treatment group of the straws for trial 2 was achieved by printing 11 (BIO4), 55 (TRI24) or 66 (OPTX24) at the beginning of the bull identification number so that AI technicians were required to note down the treatment for record keeping. However, technicians did not know the meaning of those preceding numbers. Straws were frozen using an automated freezing machine (MT Freezer 2.0, Minitube, Tiefenbach, Germany) according to the manufacturer’s instructions and stored in liquid nitrogen (−196 °C). From each batch, 10 cryopreserved samples were sent to the IFN for further laboratory analyses.

### 2.7. Laboratory Evaluations after Freezing

For laboratory evaluations, three cryopreserved straws were thawed in a water bath at 38 °C for 11 s and kept in a pre-warmed vial until further processing. Next, 50 µL of the thawed sample was mixed with 250 µL of a 1% formol–citrate solution and assessed for morphological abnormalities, as described previously [10]. Samples with >45% morphological or >30% acrosome alterations were discarded.

The remaining thawed samples were subjected to the thermo-resistance test. Hence, samples were kept in Eppendorf vials at 38 °C and progressive sperm motility was measured at 30 and 120 min incubation using CASA [10]. The threshold for progressive motility was >50% after 30 min or >40% after 120 min.

Total sperm count was assessed using NucleoCounter SP-100 (ChemoMetec, Allerod, Denmark) at 488 nm, according to the manufacturer’s instructions.

Many young bulls show a high proportion of acrosome alterations but very good sperm motility after thawing. To provide more flexibility for the assessment of ejaculates of young bulls, an index called “Total Acrosome Intact and Motile Sperm” (TAIMS) was developed. It is calculated using the following formula:

TAIMS [×10^6^/straw] = Sperm count/straw [×10^6^] × p (total sperm motility (30 min)) × p (acrosome intact sperm) (1)

and samples should not be below a preliminary threshold value of 5.25 x 10^6^/straw.

For trial 1, sperm count/straw was not measured. Therefore, the Progressively Motile and Morphologically Intact Sperm (PMMIS) was calculated:

PMMIS [%] = Progressive sperm motility (120 min) [%] × p (morphologically intact sperm).(2)

Plasma membrane and acrosome integrity was measured via flow cytometry (Cytoflex S, Beckman Coulter GmbH, Krefeld, Germany), as described previously [11]. Briefly, a master mix solution containing phosphate-buffered sodium chloride, fluorescein peanut agglutinin (FITC-PNA) and propidium iodide was incubated in the dark at 38 °C. Three straws were thawed as described above and the pooled sample was incubated at 38 °C. After 10 min as well as after 100 min of incubation, 10 µL of the pooled sample was mixed with 490 µL of the master mix solution and incubated for a further 20 min at 38 °C in the dark. Measurements were taken 30 min and 120 min after thawing and determined the percent [%] of life sperm with intact acrosome.

### 2.8. Semen Distribution and Insemination

After a quarantine of 30 days, semen portions were distributed to 54 AI technicians and used for insemination in heifers and cows between December 2020 and April 2022. AI technicians were blinded to the purpose of the study and differences among batches. Each technician received either BIO4 or treatment batches (TRI24 or OPTX24) from each bull during a certain time. Every 2 to 3 weeks, all study straws were removed from the nitrogen tanks and refilled with the opposite treatment for the bulls, ensuring that BIO4 and treatment batches (TRI24 or OPTX24) of one bull were not in the same tank at the same time, but also that each AI technician used BIO4 and treatment batches of each bull over time. Moreover, while some technicians received BIO4 batches at a certain time, others received treatment batches of those bulls and vice versa, to assure that at any time, BIO4 and TRI24 or OPTX24 batches were used. Straws were used on commercial dairy farms when the farmer or herd manager suggested an animal and it was found to be suitable for insemination (according to age or days in milk; animal was in heat; no clinical abnormalities such as metritis, endometritis, or cysts; and animal was not pregnant).

Inseminations were routinely recorded into an electronic database at the AI center and NRR60 and NRR90 were calculated per batch. Due to the preceding numbers printed on the straws for the trial (see above), it was possible to differentiate NRR for BIO4, TRI24 and OPTX24 batches.

### 2.9. Statistical Analysis

Data for trial 1 and 2 were collected in a Microsoft Excel spreadsheet (Version 2013, Microsoft Corporation, Redmond, WA, USA) and transferred into IBM SPSS for Windows (Version 26, IBM Corp, Armonk, NY, USA). Descriptive statistics and histograms were used to assess variables for normal distribution and outliers. A *p*-value < 0.05 was considered significant.

#### 2.9.1. Trial 1

Generalized linear mixed models [12] were constructed to analyze the differences between the extenders and equilibration times. Animal (bull 1-8) and ejaculate (ejaculate 1-3) were considered as random factors and cryopreservation protocol was included in the models as a fixed factor. Variance components were selected as the covariance structure. Sidak’s test (sequential) was used as a post hoc test for all models.

#### 2.9.2. Trial 2

Generalized linear mixed models were built for all outcome variables with bull and ejaculate as random effects (covariance structure = variance components) and treatment group as fixed effect. Residuals were checked for normal distribution and homoscedasticity and post hoc tests were conducted using Bonferroni correction. For models regarding NRR, we only used data where the sperm count in the straws was within 8% difference between BIO4 and treatment batches (30 BIO4 samples excluded; 2 additional samples had missing data for this variable) and number of inseminations was >50 per batch (a further 34 or 19 samples were excluded for the first inseminations or all inseminations, respectively, and nine observations did not have insemination records). We also excluded two samples from the first and total inseminations with unusually low NRR60 <50 from one bull (Bull 4). For analyses regarding the number of first inseminations and total inseminations, none of the above-mentioned restrictions were applied.

## 3. Results

### 3.1. Trial 1

Significant differences were observed among batches for progressive sperm motility at 30 and 120 min after thawing (*p* = 0.002 and *p* < 0.001, respectively, Table 2). Numerically, OPTX4, TRI24 and OPTX24 had the highest progressive motility at 30 and 120 min post thawing compared to other treatments. Batch TRI4 (50.4%) had significantly lower values compared to batches OPTX4 (58.3%) and OPTX24 (60.6%) for progressive sperm motility at 30 min post thawing. For sperm motility at 120 min post thawing, TRI4 (42.8%) had significantly lower values compared to batches BIO4, OPTX4, TRI24, and OPTX24 (51.9%, 53.7%, 52.9%, and 58.8%, respectively). The proportion of morphologically intact sperm and the proportion of acrosome defects were significantly different among groups (*p* = 0.047 and *p* = 0.002, respectively). Triladyl at 24 h equilibration time (TRI24) yielded samples with the highest proportion of morphologically intact sperm (61.9%). TRI24 (20.3%) also had the lowest proportion of acrosome defect sperm, which was significantly lower than batches BIO4 to BIO24 (25.1% to 26.4%) but not compared to OPTX24 (23.0%). At 30 min and 120 min of incubation, PMAI was lower for BIO4 and TRI4 compared to BIO24 and TRI24, respectively. For OPTX, there was a numerical increase but no significant difference between 4 h and 24 h incubation. Nevertheless, the PMAI values were highest for OPTX among the 4 h and 24 h treatments. The PMMIS was significantly different among groups (*p* < 0.001), with OPTX24 (34.2%) being significantly higher than TRI4 (25.8%) and BIO24 (28.2%).

The mean PMMIS per bull and batch ranged from 8.04% (bull 4, TRI4) to 47.63% (bull 1, TRI24), with an average of 30.72% motile and morphologically intact sperm. Based on the results from the PMMIS, 4 bulls (bulls 1, 2, 5, and 7) were selected to be used with Triladyl and 4 bulls (bulls 3, 4, 6, and 8) with OptiXcell extenders for the 24 h equilibration time (Table 3).

### 3.2. Trial 2

#### 3.2.1. Semen Evaluation

Progressive sperm motility at 30 and 120 min after thawing was significantly different among groups (*p* = 0.027 and *p* = 0.003, respectively, Table 4). For progressive motility 120 min after thawing, TRI24 (59.7%) had significantly higher values compared to BIO4 (53.0%). TRI24 (72.1%) also yielded significantly more morphologically intact sperm compared to BIO4 (67.7%), whereas OPTX24 (64.4%) yielded significantly fewer compared to BIO4. For acrosome defects, there was no difference between BIO4 and OPTX24; however, TRI24 (15.5%) had significantly fewer acrosome defects. PMAI at 30 and 120 min was significantly lower for BIO4 compared to the other two treatments (*p* < 0.001). TAIMS values were similar between BIO4 and OPTX24 (8.06 × 10^6^/straw and 7.76 × 10^6^/straw, respectively), whereas TRI24 (9.7 × 10^6^/straw) had higher TAIMS values. The final sperm concentrations in the straws were lower for TRI24 and OPTX24 (85.9 × 10^6^/mL and 83.8 × 10^6^/mL, respectively) compared to BIO4 (92.3 × 10^6^/mL, *p* < 0.001). The number of straws produced per treatment was the same (*p* = 0.341).

#### 3.2.2. Fertility

A total of 11,059 inseminations (5652 after 4 h of equilibration and 5407 after 24 h of equilibration time) were carried out between December 2020 and April 2022. Moreover, 6372 inseminations were performed on heifers and 4687 inseminations on primi- or multiparous cows. There were 6686 first inseminations (FAI) performed: 3456 after 4 h equilibration and 3230 after 24 h equilibration.

After excluding BIO4 observations with a sperm concentration with >8% difference of either TRI24 or OPTX24, observations with <50 inseminations and two unusual poor observations from bull 4, there were 3796 inseminations accounting for NRR60 and NRR90 for FAI and 7314 inseminations accounting for NRR60 and NRR90 for total inseminations (TAI).

The number of FAI or TAI per ejaculate did not differ among treatment groups (*p* = 0.215 and *p* = 0.197, respectively). Furthermore, there was no difference in NRR60 or NRR90 for FAI or TAI among treatment groups (*p* > 0.08, Table 5).

## 4. Discussion

This study investigated the effect of prolonged equilibration time on in vitro sperm parameters and of using a bull-specific extender with prolonged equilibration time on field fertility in Holstein Friesian cattle. Other studies commonly used the same extenders for all bulls [6,7,13]. Different researchers [13,14] tested but did not find significant interactions between bull and extender type. Therefore, the aforementioned authors found that changes in laboratory parameters or fertility with different extenders did not depend on the bull used. In contrast, Salman et al. [15] found that the change with different equilibration times significantly depended on the individual bull. To our knowledge, using a bull-specific extender for 24 h equilibration time in our study was a new approach. It was based on the anecdotal observation that sperm from some bulls yielded better laboratory results with certain extenders compared to other extenders. Interestingly, there was an equal number of bulls showing numerically better results with either TRI24 or OPTX24 in trial 1, resulting in the same number of bulls being assigned to treatments TRI24 and OPTX24 in trial 2. To date, it is unclear what factors caused these individual differences in cryopreservation characteristics with different semen extenders. A recent study by Jakop et al. [10] found differences among bulls in radical reduction capacity and lipid alteration during cryopreservation of sperm. They suggested that it might be useful to implement individualized extenders for certain bulls with poor cryopreservation characteristics. Batches TRI4, OPTX4 and BIO24 of our study yielded generally poorer results and, therefore, they were not used in trial 2. While we could demonstrate that using bull-specific extenders was beneficial in terms of achieving better laboratory results for each bull (see trial 1), we were not able to differentiate whether this approach helped improve field fertility for the 24 h equilibration time with our study.

Similar to other studies [5,6,7], we found overall no difference or better laboratory results with 24 h equilibration time in trial 1 (TRI24 and OPTX24) and trial 2 compared to a short equilibration of 4 h. However, these other studies commonly used the same extender for the different equilibration times, whereas our trial 2 used a different extender for the 4 h (BioXcell) and the 24 h (Triladyl or OptiXcell) equilibration time. Therefore, with trial 2, we cannot differentiate whether the differences found between 4 h and 24 h equilibration time were due to differences in equilibration times or extenders or both. Moreover, the extenders Triladyl and OptiXcell, both with 24 h equilibration times in trial 2, should only cautiously be compared directly because there were different bulls in each group. For trial 1, differences among groups can be attributed to either extenders or equilibration times due to the different split-sample design.

For TRI24, there were superior results regarding progressive sperm motility after 120 min incubation, acrosome defects, PMAI and PMMIS compared to TRI4 in trial 1. This is in agreement with Fleisch et al. [7], who found numerically higher sperm motility directly after thawing and 180 min after thawing and significantly higher PMAI when comparing 24 h to 4 h equilibration time with Triladyl.

Our results were statistically not different for progressive motility, morphology and PMMIS for BIO24 and OPTX24 compared to BIO4 and OPTX4, respectively (trial 1). For BIO, PMAI at 30 min and 120 min of incubation was significantly higher with the longer incubation time. Shahverdi et al. [3] also did not find differences in sperm motility, kinematic sperm parameters or DNA damage when using BioXcell for 4 h or 16 h in buffalo bull semen. Contrarily, they did not find differences in PMAI with different incubation times. Similar to our study, Fleisch et al. [7] found only numerically better sperm motility when using OptiXcell for 24 h compared to 4 h equilibration. Others found a small decrease in progressive motility directly after thawing, but not after 4 h of incubation, when using 24 h equilibration compared to 4 h with OptiXcell [15]. The aforementioned authors also found significantly better plasma membrane integrity [7] and chromatin structure with no effects on oxidative stress, or on apoptotic or capacitation markers [15].

In our study, we used different equilibration conditions for the 4 h and 24 h equilibration times at 4 °C. While the short-time equilibration was carried out in straws on racks, the 24 h equilibration was performed in cups and thereafter, sperm was filled into straws at 4 °C. Tirpan et al. [16] did not find differences in sperm motility and PMAI when comparing these two different equilibration conditions to each other while using the extender AndroMed (Minitube, Tiefenbach, Germany).

Nevertheless, there were no differences in field fertility when comparing different equilibration times and extenders in the present study. These results are also in agreement with other studies [5,6,7]. This might be related to high sperm concentrations per straw used for commercial inseminations that compensate for minor changes in laboratory sperm parameters [7,17]. Christensen et al. [17] also commented that preselection of bulls with good fertility and ejaculates with good motility and morphology likely further reduces the ability to detect differences in field fertility among treatments. The same authors found in their large study that 95% of the variability of NRR56 could not be explained by effects such as herd, AI technician, bull, or ejaculate. Therefore, in the current commercial AI system that utilizes the aforementioned procedures such as high sperm count per insemination, preselection of bulls and pre-screening of ejaculates, only a minor portion of the variability of NRR is explained by sperm characteristics. Consequently, it is very difficult to observe differences in field fertility, even with very large trials. Amann and Hammerstedt [18] suggested that it might be more appropriate, although unpractical, to conduct reproductive field trials with much lower sperm numbers (i.e., 0.8 to 1.5 × 10^6^) than necessary for optimal fertility to observe even small differences between treatments. In contrast, in the present study, the AI center aimed for at least 10 × 10^6^ motile sperm per straw after thawing to obtain optimal reproductive results. On the other hand, it might be that some laboratory parameters were affected by differences in extender properties and equilibration times but that there were only minor real differences in sperm quality and field fertility related to extenders and equilibration times. For example, there appears to be a different utilization of glycerol when an egg-yolk-based extender is used compared to milk-based extenders [19]. Similarly, one could hypothesize that incorporation of stains that are used to assess viability or plasma membrane integrity could be affected by different extender properties. Moreover, egg yolk particles originating from the extender might affect flow cytometric measurements [20].

Additionally, there were limitations in trial 2 of our study regarding the sperm concentrations in the straws and the number of inseminations per batch and ejaculate. Likely, there were differences in sperm concentrations in the straws because BIO4 straws were diluted using an electrical pump, whereas TRI24 and OPTX24 were diluted conventionally by hand. The pump was calibrated regularly, and the volume of extender used was re-checked after filling. Thus, it is unclear what caused this imprecision. To avoid confounding of fertility results due to different sperm concentrations, we excluded BIO4 samples with sperm concentrations that were >8% different from either TRI24 or OPTX24. When applying this threshold of 8%, there were no significant differences in sperm concentrations in the straws among groups. Moreover, we only included observations where the NRR was calculated based on >50 inseminations. This procedure improved the precision of the NRR measurements, as it allowed us to measure NRR with a maximum width of the 95% confidence interval (95% CI) of 0.26 for an NRR of 0.7 and a maximum width of the 95%CI of 0.28 for a NRR of 0.6 [21]. Ultimately, only 41% and 54% of observations were used for statistical analysis of NRR for FAI and TAI, respectively. This could have led to insufficient power to detect differences in field fertility among groups in our study.

## 5. Conclusions

In vitro sperm parameters, including progressive sperm motility at 120 min, acrosome defects and PMAI were superior for samples equilibrated with Triladyl for 24 h compared to 4 h. When using BioXcell, 24 h equilibration improved PMAI values compared to 4 h equilibration. Otherwise, samples equilibrated using BioXcell or OptiXcell for 24 h yielded similar results to 4 h equilibration with the same extenders. However, using bull-specific extenders and a prolonged equilibration time of 24 h did not affect field fertility. Nevertheless, it would be possible to change from 4 h to 24 h equilibration time, when using Triladyl or OptiXcell for the longer equilibration time, without impairing field fertility.

## Figures and Tables

**Table 1:** Description of Holstein Friesian bulls used in trial 1 and 2. Age and overall breeding value are changing over time and correspond to the beginning of trial 1.

Bull	Color	Age	Overall Breeding Value	No. Ejaculates in Trial 2	No. Inseminations in Trial 2
1	BW	3.83	146	7	1845
2	BW	2.75	160	8	2464
3	BW	2.58	144	6	653
4	BW	1.75	155	8	1498
5	BW	3.50	141	10	859
6	BW	3.33	153	5	613
7	RH	2.83	160	7	1652
8	RH	1.67	144	9	1473

BW = black and white Holstein; RH = red Holstein.

**Table 2:** Predicted means for laboratory parameters of cryopreserved sperm using different extenders and equilibration times in 24 ejaculates from eight Holstein Friesian bulls (three ejaculates each) in trial 1 (*n* = 144).

Parameter (%)	Batch *						SEM	*p*-Value
	BIO4	TRI4	OPTX4	BIO24	TRI24	OPTX24		
Prog. motility (30 min, %)	56.0 ^a,b^	50.4 ^b^	58.3 ^a^	55.2 ^a,b^	57.3 ^a,b^	60.6 ^a^	3.123	*p* = 0.002
Prog. motility (120 min, %)	51.9 ^b,c^	42.8 ^a^	53.7 ^b,c^	49.5 ^a,b^	52.9 ^b,c^	58.8 ^c^	3.379	*p* < 0.001
Morph. intact sperm (%)	59.2	58.0	57.0	56.7	61.9	58.0	2.583	*p* = 0.047
Acrosome defects (%)	25.1 ^a^	26.4 ^a^	25.8 ^a^	25.9 ^a^	20.3 ^b^	23.0 ^a,b^	1.788	*p* = 0.002
PMAI ** 30 min (%)	49.5 ^a^	52.1 ^a^	61.4 ^b^	59.0 ^b^	61.7 ^b^	62.9 ^b^	1.984	*p* < 0.001
PMAI 120 min (%)	48.4 ^a^	50.6 ^a,b^	58.1 ^c,d^	54.4 ^b,c^	56.7 ^c,d^	59.8 ^d^	1.978	*p* < 0.001
PMMIS (%)	31.2 ^a,c^	25.8 ^a^	31.6 ^b,c^	28.2 ^a,b^	33.3 ^b,c^	34.2 ^c^	2.726	*p* < 0.001

* Treatments: BIO4 = BioXcell at 4 h equilibration time, TRI4 = Triladyl at 4 h equilibration time, OPTX4 = OptiXcell at 4 h equilibration time, BIO24 = BioXcell at 24 h equilibration time, TRI24 = Triladyl at 24 h equilibration time, OPTX24 = OptiXcell at 24 h equilibration time, ** PMAI = plasma membrane and acrosome integrity, PMMIS = progressively motile and morphologically intact sperm, *n* = count, Prog. = Progressive, ^a, b, c, d^ different letters denote significant differences at *p* < 0.05.

**Table 3:** Mean (standard deviation) for progressive motility (120 min) × p (morphologically intact sperm) in cryopreserved sperm using three extenders (BioXcell, Triladyl, OptiXcell) and 4 h or 24 h equilibration time for eight Holstein Friesian breeding bulls (three ejaculates in each batch per bull) from trial 1. The ideal extender for trial 2 for each bull was chosen based on the highest result at 24 h equilibration time (marked in bold).

Bull	Batch *						Overall	Chosen Extender
	BIO4	TRI4	OPTX4	BIO24	TRI24	OPTX24		
1	34.13 (1.83)	35.00 (10.08)	43.21 (8.10)	22.68 (4.33)	**47.63 (2.02)**	42.61 (4.70)	37.54 (9.78)	Triladyl
2	29.13 (9.21)	29.08 (2.12)	26.50 (4.42)	20.12 (4.51)	**32.56 (8.92)**	30.37 (4.25)	27.96 (6.57)	Triladyl
3	34.02 (7.78)	29.21 (8.25)	39.79 (0.54)	33.04 (9.19)	34.48 (10.72)	**39.72 (7.77)**	35.04 (7.78)	OptiXcell
4	28.61 (15.53)	8.04 (8.13)	27.77 (11.52)	25.42 (7.62)	22.20 (12.51)	**25.76 (5.98)**	22.97 (11.52)	OptiXcell
5	31.49 (9.88)	27.63 (7.55)	28.81 (4.91)	34.98 (11.53)	**41.31 (6.64)**	34.47 (7.99)	33.11 (8.44)	Triladyl
6	37.98 (2.66)	35.68 (14.48)	37.31 (4.12)	37.07 (3.32)	33.91 (3.20)	**41.94 (3.44)**	37.32 (6.15)	OptiXcell
7	36.96 (3.96)	28.54 (5.13)	32.91 (3.66)	32.12 (2.29)	**34.35 (1.89)**	32.95 (4.10)	32.97 (4.03)	Triladyl
8	17.41 (6.59)	13.34 (12.31)	16.43 (12.85)	19.88 (6.06)	20.06 (12.87)	**25.90 (6.09)**	18.84 (9.30)	OptiXcell

* Treatments: BIO4 = BioXcell at 4 h equilibration time, TRI4 = Triladyl at 4 h equilibration time, OPTX4 = OptiXcell at 4 h equilibration time, BIO24 = BioXcell at 24 h equilibration time, TRI24 = Triladyl at 24 h equilibration time, OPTX24 = OptiXcell at 24 h equilibration time.

**Table 4:** Predicted means (standard error of the mean) for laboratory parameters of cryopreserved sperm for split samples (BIO4 and TRI24 or BIO4 and OPTX24, depending on the bull) from eight bulls and five to 10 ejaculates per bull (*n* = 60 ejaculates).

Parameter		Treatment *		*p*-Value
	BIO4	TRI24	OPTX24	
Prog. motility 30 min (%)	60.3 (1.87)	64.3 (2.18)	63.0 (2.24)	0.027
Prog. motility 120 min (%)	53.0 ^a^ (1.54)	59.7 ^b^ (2.00)	56.6 ^a,b^ (2.10)	0.003
Morph. intact sperm (%)	67.7 ^b^ (1.76)	72.1 ^c^ (1.88)	64.4 ^a^ (1.90)	<0.001
Acrosome defects (%)	20.6 ^b^ (0.89)	15.5 ^a^ (1.04)	20.3 ^b^ (1.08)	<0.001
PMAI ** 30 min (%)	57.9 ^a^ (1.03)	69.7 ^c^ (1.31)	60.6 ^b^ (1.37)	<0.001
PMAI 120 min (%)	55.1 ^a^ (0.95)	65.2 ^b^ (1.17)	63.1 ^b^ (1.21)	<0.001
TAIMS ** (×10^6^/straw)	8.06 ^a^ (0.35)	9.70 ^b^ (0.42)	7.76 ^a^ (0.43)	<0.001
Final sperm concentration *** (× 10^6^/mL)	92.3 ^b^ (2.76)	85.9 ^a^ (3.01)	83.8 ^a^ (3.04)	<0.001
Number of straws	194 (13.5)	197 (14.1)	202 (14.2)	0.341

* Treatments: BIO4 = BioXcell at 4 h equilibration time, TRI24 = Triladyl at 24 h equilibration time, OPTX24 = OptiXcell at 24 h equilibration time, ** PMAI = plasma membrane and acrosome integrity, TAIMS = total acrosome intact and motile sperm, *** Final sperm concentration after dilution in insemination straws, Prog. = Progressive, ^a, b, c^ letters denote differences at *p* < 0.05.

**Table 5:** Field fertility (number of inseminations and non-return rates) from eight Holstein Friesian bulls comparing BIO4 and either TRI24 or OPTX24 in a split-sample procedure.

Parameter	N Observations	N Inseminations		Treatment *		*p*-Value
			BIO4	TRI24	OPTX24	
Count FAI **	111	6685	62.3 (10.36)	54.1 (10.84)	46.0 (9.49)	0.215
NRR60 FAI (%)	45	3796	71.3 (1.46)	68.2 (1.40)	67.2 (2.01)	0.088
NRR90 FAI (%)	45	3796	65.4 (1.75)	63.2 (1.78)	61.6 (2.33)	0.269
Count TAI **	111	11,056	104.0 (13.77)	96.3 (15.12)	87.2 (15.34)	0.197
NRR60 TAI (%)	60	7314	67.3 (1.69)	64.9 (1.76)	67.8 (1.95)	0.336
NRR90 TAI (%)	60	7314	61.8 (1.79)	60.1 (1.87)	62.5 (2.07)	0.582

* Treatments: BIO4 = BioXcell at 4 h equilibration time, TRI24 = Triladyl at 24 h equilibration time, OPTX24 = OptiXcell at 24 h equilibration time, ** FAI = first artificial inseminations, TAI = total artificial inseminations.

## Data Availability

Data will be made available upon request.
